# Editorial: How Do Motivational States Influence Motor Resonance?

**DOI:** 10.3389/fnhum.2020.00027

**Published:** 2020-02-14

**Authors:** Cosimo Urgesi, Kaat Alaerts, Laila Craighero

**Affiliations:** ^1^Laboratory of Cognitive Neuroscience, Department of Languages and Literatures, Communication, Education and Society, University of Udine, Udine, Italy; ^2^Scientific Institute, IRCCS E. Medea, Bosisio Parini, Italy; ^3^Research Group for Neuromotor Rehabilitation, KU Leuven, Leuven, Belgium; ^4^Department of Biomedical and Specialty Surgical Sciences, University of Ferrara, Ferrara, Italy

**Keywords:** action observation, imagery, motivational state, motor resonance, top-down modulation

Viewing or imagining actions triggers an activation of the observer's motor system that overlaps the representations of executed actions (Rizzolatti and Craighero, 2004). This “vicarious” motor activation has been referred to as *motor resonance* and it can be directly gauged by recording brain activity in the observer or imaginer, for example by recording with electroencephalography (EEG) a suppression of the mu rhythm from central scalp regions (Pineda, 2005) or by recording motor evoked potentials (MEPs) in response to transcranial magnetic stimulation (TMS) of the primary motor cortex (Fadiga et al., 2005). Furthermore, it can be also indirectly assessed by considering the influence that the action has on its execution or evaluation (e.g., facilitated mimicry of corresponding actions, but motor interference during the simultaneous observation and execution of incompatible actions; Craighero et al., 2002, 2014) or the influence that action observation has on the execution of similar actions (e.g., during observational learning; Urgesi et al., 2012). Using these methods, many experiments have shown that motor resonance occurs in a muscle-specific fashion according to somatotopic rules (Urgesi et al., 2006; Alaerts et al., 2009), that it is time-locked to the movement phases (Borroni et al., 2005; Alaerts et al., 2012), and that it follows the pattern of facilitation or inhibition of motor activity involved in selecting or refraining from performing a particular action (Romani et al., 2005; Schütz-Bosbach et al., 2009; Alaerts et al., 2010a). Based on these somatotopic, time-locked and direction-specific features, motor resonance has been commonly considered to reflect a fine-grained encoding of action kinematic aspects (Naish et al., 2014). This motor replica may support action perception and conception, since the automatically induced, sensorimotor representation of the perceived or imagined action corresponds to what is spontaneously generated during action execution and whose outcome is known to the agent.

However, even if a butcher knows exactly how to use a knife to slaughter a cow he probably does not know what it really means to use the same knife to infer a fatal stab to a person: motivations and consequences are totally different. As well, watching someone devouring a greasy hamburger can arouse envy or disgust depending on the level of satiety of the observer or on his diet. Therefore, if mapping others' actions onto one's own sensorimotor representation cues the goal and, possibly, the ultimate intention of the agent, motor resonance must necessarily encode also aspects not simply related to the kinematics of the movement.

This Research Topic includes original research and review contributions aimed at assessing which cognitive processes and neural mechanisms are involved in exerting a top down modulation of motor resonance according to stimulus features and task requirements, as well as according to actor's and observer's motivational states.

Amoruso and Finisguerra provided a comprehensive overview of the various pieces of evidence that have challenged the view of an automatic motor resonance by showing that motor resonance is pervious to top-down modulation. In particular, by examining TMS studies that have measured modulation of MEPs amplitudes during action observation, they have shown that, when the experimental stimuli stop displaying only aseptic moving body parts, but include more complex information on the actor, object and environment, motor resonance goes beyond the low-level mapping of the observed kinematics. In fact, it may be shaped by the intended goal (Cattaneo et al., 2009; Alaerts et al., 2010b; Senot et al., 2011; Finisguerra et al., 2015), the underlying intention (Tidoni et al., 2013; Amoruso and Urgesi, 2016b; Craighero and Mele, 2018; Finisguerra et al., 2018) and/or the embedding context (Amoruso and Urgesi, 2016a; Amoruso et al., 2016, 2018) of observed actions. Accordingly, the empirical contribution of Rens and Davare found that observing object lifting movements improved the force scaling of successively performed lifting movements, since observing the kinematics differences between actions directed to objects of different weights updates internal sensorimotor representations and anticipatory motor control. Crucially, however, these effects were modulated by the situational context in which the actions took place, with greater effects when the model actor intended to commit an error in object lifting than when he intended to perform skillful movements.

Moving to more direct measures of motor resonance, Karakale et al. reported that suppression of the mu rhythm recorded from central electrodes was modulated by the emotional content of observed facial gestures, with greater suppression for biological than non-biological stimuli only for neutral movements void of any emotional meaning (i.e., mouth opening-closing movements), but not for emotional (i.e., happy or sad) expressions. In a similar vein, Maegherman et al. failed to find an amplitude increase of MEPs evoked by TMS in finger and facial muscles during imagery of simple squeezing movements involving the fingers or the lips, respectively. This finding contrasts with previous evidence of imagery-related facilitation of the motor cortex for more complex movements (e.g., Fourkas et al., 2006, 2008), and suggests that the recruitment of the primary motor cortex during imagery is conditional on task difficulty and requirements. Finally, the contribution of Farwaha and Obi further supported top-down modulation of motor resonance by highlighting the correlation between the degree of motor resonance during action observation and the online status of the observer. They found that individuals who have fewer Instagram followers than they follow (i.e., followers) show greater motor resonance than individuals who have more followers than they follow (i.e., leaders). This finding converges with similar evidence that observer's sense of power (Hogeveen et al., 2014) or socioeconomical status (Varnum et al., 2016) in the real world also affects motor activation during action observation and supports the flexible nature of motor resonance according to not only external stimulus and task complexity, but also internal observer's factors.

In conclusion, this Research Topic has collected initial evidence supporting the hypothesis that motor resonance can be modulated by actor's and observer's intentions, needs, values, emotions, and attitudes. However, further studies are required to clarify which cognitive processes and neural mechanisms are involved in exerting this top-down modulation.

## Author Contributions

CU wrote the first draft of the manuscript. KA and LC revised it critically. All authors approved the submitted version.

### Conflict of Interest

The authors declare that the research was conducted in the absence of any commercial or financial relationships that could be construed as a potential conflict of interest.
